# Incorporation of Cellulose-Based Aerogels into Textile Structures

**DOI:** 10.3390/ma17010027

**Published:** 2023-12-20

**Authors:** Sebnem Sozcu, Mohanapriya Venkataraman, Jakub Wiener, Blanka Tomkova, Jiri Militky, Aamir Mahmood

**Affiliations:** Department of Material Engineering, Faculty of Textile Engineering, Technical University of Liberec, 46117 Liberec, Czech Republic; jakub.wiener@tul.cz (J.W.); blanka.tomkova@tul.cz (B.T.); jiri.militky@tul.cz (J.M.); aamir.mahmood@tul.cz (A.M.)

**Keywords:** bio-based aerogel, multifunctional properties, thermal insulation, flame retardant, textile applications

## Abstract

Given their exceptional attributes, aerogels are viewed as a material with immense potential. Being a natural polymer, cellulose offers the advantage of being both replenishable and capable of breaking down naturally. Cellulose-derived aerogels encompass the replenish ability, biocompatible nature, and ability to degrade naturally inherent in cellulose, along with additional benefits like minimal weight, extensive porosity, and expansive specific surface area. Even with increasing appreciation and acceptance, the undiscovered possibilities of aerogels within the textiles sphere continue to be predominantly uninvestigated. In this context, we outline the latest advancements in the study of cellulose aerogels’ formulation and their diverse impacts on textile formations. Drawing from the latest studies, we reviewed the materials used for the creation of various kinds of cellulose-focused aerogels and their properties, analytical techniques, and multiple functionalities in relation to textiles. This comprehensive analysis extensively covers the diverse strategies employed to enhance the multifunctionality of cellulose-based aerogels in the textiles industry. Additionally, we focused on the global market size of bio-derivative aerogels, companies in the industry producing goods, and prospects moving forward.

## 1. Introduction

Aerogels are fascinating substances of the twenty-first century due to their unique structure [1]. They possess remarkable properties like high porosity, low density, huge surface area, and superb heat and sound insulation. However, their low mechanical strength and high production costs restrict their usefulness [2].

Aerogels are extremely porous nanostructured materials invented by Kistler in 1931 [2,3]. Aerogels may be created using either supercritical drying or freeze-drying processes. The material’s microporous structure stays intact throughout drying in both circumstances. Kistler’s first aerogel, prepared by supercritical drying, was silica-based. During the creation of silica aerogels, toxic precursors were used, and the aerogels formed were not biodegradable [4]. The costly process of making this type of aerogel, with the restrictions described earlier (non-biodegradable and toxic precursors), confirms the restricted use of this material [4].

Aerogels are also used to develop a wide range of tools, such as in optoelectronics, adsorption catalysis, sound insulation, pharmaceutical materials, and aerospace materials [5,6,7,8,9,10]. However, some drawbacks, together with the huge expenses associated with their fabrication, have severely limited the use of aerogels [11]. Aerogels can be formed using a wide range of materials, including inorganic ones [12], synthetic polymer-based materials [13], and natural polymer-based materials [14,15] such as cellulose [16,17], depending on the starting substance used for their manufacture [2,18]. Aerogels, which are praised for being among the extraordinary materials of the twenty-first century due to their outstanding mechanical properties and extremely low weight, have gained attention as “miracle materials”. These aerogels have drawn a lot of interest from researchers in a variety of sectors due to their remarkable endurance under harsh working conditions [19]. 

Cellulose aerogels, in particular, have cellulose’s renewability, biocompatibility, and biodegradability, as well as additional benefits like low density values (0.0005–0.35 g·cm^−3^), enhanced porosity (84.0–99.9%), and a huge specific surface area, representing the materials with the highest potential in the 21st century [11]. Cellulose aerogels possess greater compressive strength (0.0052–16.67 MPa) and superior biodegradability [11]. So, cellulose aerogels are eco-friendly and versatile modern materials with enormous opportunities for application in adsorption and oil/water separation, heat separation, biomedical materials, metal nanoparticle/metal oxide carriers, and carbon aerogel precursors, and they possess high reusability, thereby minimizing economic losses. Moreover, the notable advantage of bio-based aerogels lies in their natural decomposition, avoiding additional environmental harm. Drawing on knowledge from the body of literature already in existence, future studies related to bio-based aerogel absorbents may center on evaluating their stability in harsh conditions. This strategy could lead to improvements in the development of more effective solutions, especially for dealing with oil spills, which would eventually reduce financial losses [20].

Cellulose aerogels, as porous solids, are typically produced through a two-step process: Cellulose or cellulose derivatives are dissolved/dispersed, forming a cellulose sol using the sol–gel method. Subsequently, the cellulose sol is dried by preserving the sol’s three-dimensional porous structure [11].

This study describes the materials used in the production of many types of cellulose-based aerogels, their features, analytical techniques, and their multifunctionality with respect to textiles. For the textiles sector, the different techniques for the multifunctionality of cellulose-based aerogels and analyses are comprehensively discussed [11].

## 2. Creating Cellulose Aerogels

Cellulose can be derived from various sources [21,22,23], such as plants or plant-based materials, e.g., rice straw. It can also be derived from the most commonly used sources [24], such as cannabis [25], cotton [26,27], wood [28,29], potato tubers [30], coconut (coir) [31], and bagasse [32]. The extraction of cellulose involves obtaining it from specific plant species, employing various production processes, including pretreatment, post-treatment, and disintegration processes, which determine its performance characteristics, like size, molecular chain length (degree of polymerization (DP)), thermal stability, and degree of crystallinity [33,34]. As a result, the plant source significantly impacts the structure and performance of cellulose aerogels [34,35].

Cellulose is a member of the polysaccharide family, which are the primary building elements for plants. Plants are the first or most basic link in the food chain (which details the feeding interactions of all living organisms) [35,36,37]. Cellulose is a key component of many natural fibers, including cotton and other plants [38,39].

Cellulose exhibits insolubility in water and the majority of common solvents [36], owing to strong intramolecular and intermolecular hydrogen bonding between individual chains [35]. Cellulose is employed in a variety of products despite its poor solubility, including composites, netting, upholstery, coatings, packaging, and paper. To make cellulose more processable and to produce cellulose derivatives, which can be customized for certain industrial purposes, cellulose is chemically modified [40,41].

**Figure 1:**
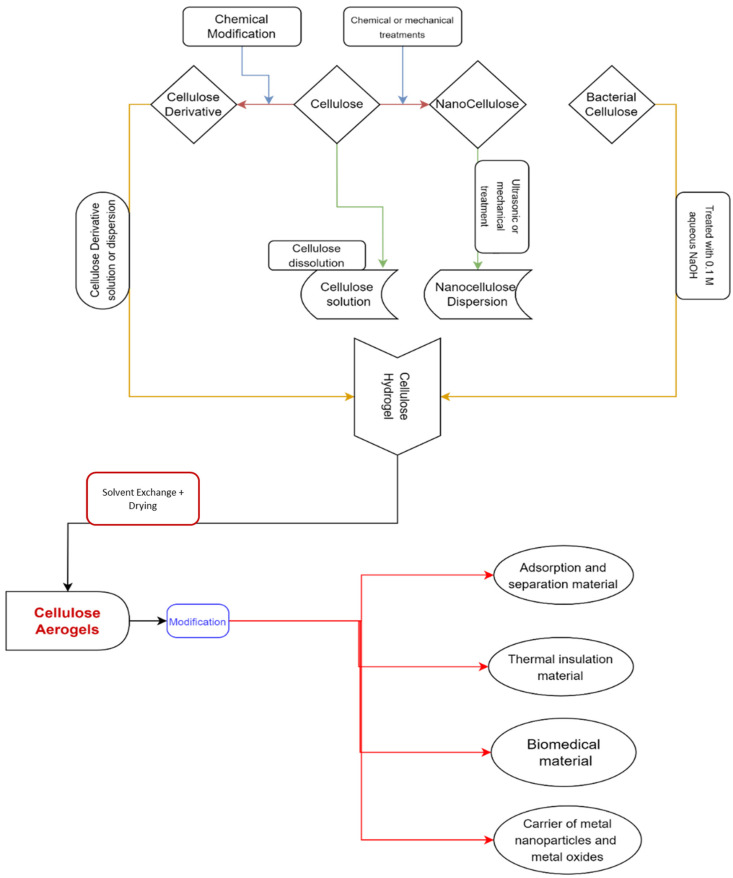
A graphical representation depicting the manufacture and application of cellulose aerogels [53] (permission was granted by the authors, Sebnem Sozcu et al.; internal publication in the Technical University of Liberec, ISBN 978-80-7494-607-3).

Aerogel materials can benefit from the mechanical qualities and moisture affinity of cellulose and its derivatives [42,43,44]. To keep their solid network, the cellulose is dissolved in appropriate media, such as NMMO, hydrates of some molten salts, and ionic liquids. Then drying processes such as supercritical drying or freeze-drying [45,46,47,48,49] are used for the preparation of cellulose aerogels. There are several strategies for creating cellulose aerogels that have been published in the literature [46,47,50,51,52]. The manufacturing of cellulose aerogels and their applications are depicted in Figure 1.

Furthermore, employing cellulose as a precursor in the production of aerogels has the following benefits: (1) The supply of cellulose raw materials is infinite and renewable. (2) Because the cellulose chain contains a lot of hydroxyl groups, no crosslinking agent is needed during the aerogel production process. A stable three-dimensional (3D) network structure can be created by employing hydrogen bond physical crosslinking both within and between molecules, making the aerogel production technique incredibly easy. (3) Chemical cellulose modification is a quick and easy method of enhancing the structural integrity and mechanical strength of cellulose aerogels. The performance and concentration of the cellulose have a significant impact on the manufacturing process and structural characteristics of cellulose aerogels [11]. To create cellulose gels, the cellulose morphology and the structure of the cellulose fibers must be changed. This is done by using a suitable solvent [54,55,56] that is capable of breaking the large hydrogen bonding network that does not degrade along the cellulose polymer chain, or by beginning polymer chain derivation processes [46].

Because of their numerous interior pores and effective heat insulation, cellulose aerogels are among the most capable thermal insulation materials for construction or domestic applications (for example, refrigerator insulation materials) and have the potential to improve their poor properties, such as flame retardancy, huge swelling, and antibacterial properties [57,58]. Regarding the cellulose aerogel’s properties, starting materials, type used in synthesis and their usage field has been discussed in Table 1.

### 2.1. Sol–Gel Procedure

**Figure 2:**
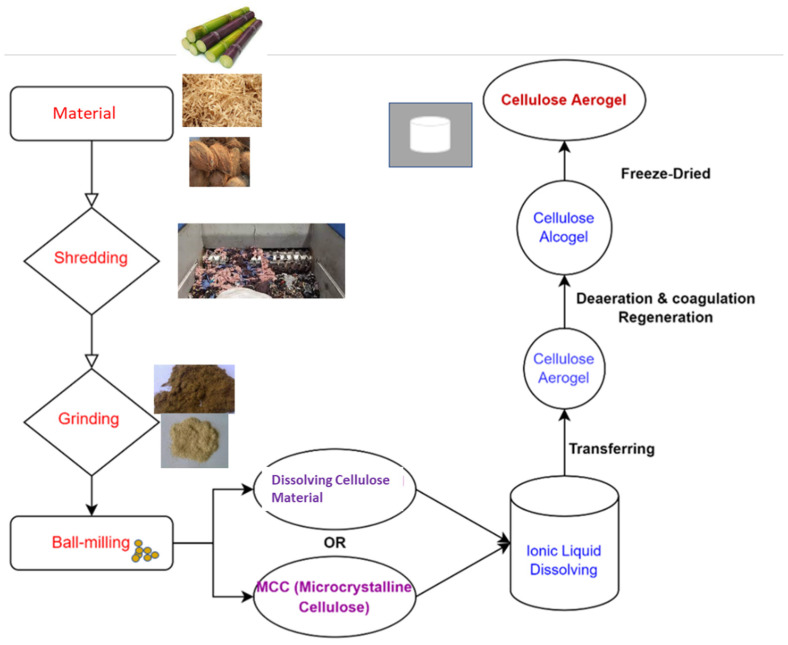
A schematic diagram depicting the cellulose aerogel preparation process [53].

As shown in Figure 2, all steps of the production process influence the gel structure, determining its characteristics and, as a result, its utilization [68]. Not only the material type used to prepare the cellulose aerogel, the agent used to dissolve the cellulose material, the type of drying method, etc., but also other techniques, as listed below, are frequently employed in improving the structural attributes and characteristics of cellulose-based gels [69].

- A colloidal suspension is produced by dispersing solid nanoscale particles formed from a reactant in a liquid.
- Adding an acidic or basic catalyst initiates crosslinking and leads to the linkage and spreading of particles, forming an interlinked network configuration.
- Gel aging: to strengthen the gel’s backbone and material toughness, it is aged in its mother solution.
- To avoid gel fractures, the solvent is extracted from the pores of the gel during drying. [70].

The procedure starts with the creation of a colloidal solution, often known as a sol. A solution of reactants and solvents contains solid nanoparticles or initiator materials.

In the course of this procedure, the introduction of a catalyst aids in polymerization, involving hydrolysis and polycondensation chemical reactions. This leads to the formation of crosslinks and branches between polymeric species, giving rise to the creation of a three-dimensional porous network within a wet, gel-like structure before aging as mentioned in the bullet points above for creating aerogels [70,71]. 

Sol–gel products may be manufactured from a variety of substances, including oxides (such as silicon dioxide and oxide minerals), natural compounds (such as large molecules like plant-derived materials), and carbon-based substances (such as 2D carbon allotropes and carbon nanopipes) [70,72]. In this study, pineapple-fiber (PF) aerogels were successfully created by pretreating PFs with naturally decomposable polyvinyl alcohol (PVA). The PVA solution preparation was combined with PFs and freeze-dried. According to the findings, the PFs have high porosities (~99%), ultralow densities, and microporous formations, as shown by field-emission scanning electron microscopy, Brunauer–Emmett–Teller isotherm, and X-ray diffraction analysis. The exceptionally low thermal conductivity of the PF aerogel demonstrated its applicability for thermal barrier uses. A thermal coat wrapped over a water bottle with a PF aerogel filling can unquestionably keep the water temperature near 0 °C (just above the freezing temperature) for up to 6 h (initial temperature: −3 °C) and above 40 °C for up to 2.5 h (initial temperature: 90 °C). The thermal coat has a potential thermal barrier that is nearly three times that of a product that is currently on the market [73]. In another study, the goal was to develop a thermal coat for army canteens based on a paper waste cellulose aerogel to increase the life of ice slurry for dynamic army troops in exercises or operations. However, because of the minimal stretching capacity and the ease with which the bio-based aerogel structure can be damaged, the bio-based aerogel must be sandwiched between two protective layers to make the thermal coat more durable. The paper waste was combined with deionized water and crosslinked with *Kymene* chemicals (crosslinkers based on polyamide-epichlorohydrin resin) before being frozen overnight. After freezing, the gel was dried using the lyophilization drying technique at −91 °C to create cellulose aerogels, followed by the crosslinking process in the dryer for 3 h at 120 °C. Following all measurements, the results showed that the heat barrier function of the developed thermal coats was significantly better than that of marketed thermal flasks and similar to that of vacuum flasks for the same duration of 4 h and the same surrounding temperatures [74]. Cellulose aerogels were made from dissolvable cellulose filaments in melts of calcium thiocyanate salt hydrate in this study, followed by regenerating in ethanol and drying under supercritical CO_2_. It is possible to create uniform-structured bio-based aerogels with minimal bulk mass. The microstructure of bio-based aerogels exhibited a continuous 3D network with a large specific surface ratio coupled with a significantly sponge-like structure (up to 98%). This research enabled the examination of increased cellulose amounts of up to 6 wt%. Bio-based aerogels displayed remarkable physical strength and heat transfer efficiency for textile applications at atmospheric pressure. Moreover, the Young’s modulus of cellulose aerogels showed that it can be reached at 13.5 MPa, while the Poisson ratio was near zero [75]. Yangyang exploited discarded cotton textiles to enhance the anti-flaming capabilities of cellulose aerogels by producing magnesium hydroxide nanoparticles in situ in cellulose gel nanostructures. In addition, three-dimensional nanoporous cellulose gels were produced by disintegrating and coagulating cellulose in an aqueous NaOH/urea solution, and these were employed as patterns for the unclustered production of magnesium hydroxide nanoparticles. According to the findings, the produced mixture–matrix aerogels have extremely porous architectures and exceptional thermal isolation characteristics with minimal heat transfer. In addition, effective flame-retardant and mechanical characteristics were obtained [76].

The sol–gel process is linked to the organic polymer type. Because the molecular composition of organic polymer variants contains a restricted amount of active (e.g., hydroxyl) groups, a connecting agent is often necessary to achieve the required gel structure [11]. 

The creation of bio-based aerogels from nanoscale crystalline polysaccharides and dissolvable organic polymers from various materials is shown schematically in Figure 2, achieved simply by recovering them as a coagulant from their liquid solution, followed by lyophilization and the resulting regenerated cellulose aerogel [77].

### 2.2. Drying Methods of Cellulose-Based Aerogels

The most crucial phase in the manufacture of aerogels is drying. The drying process influences the shape of cellulose aerogels. Due to the capillary pressure, traditional drying processes can result in the collapse of the gel pore structure. Supercritical drying (using alcohol, acetone, or CO_2_) and vacuum freeze-drying are extensively used for cellulose aerogel manufacturing procedures [78,79]. The sublimation of a solid, such as frozen water, from a moist precursor’s pores is identified as freeze-drying. As a result of the formation of ice during the process of water freezing, freeze-drying produces a sheet-like cellulose network with large and linked holes with a width of numerous micrometers [79] Under supercritical (sc) conditions, the absence of a liquid/gas meniscus results in the complete elimination of surface tension between the liquid and gas phases. ScCO_2_ dried aerogels usually have a cauliflower-like cellulose arrangement, which is an assemblage of tiny shaggy beads.

#### 2.2.1. Drying with Supercritical Carbon Dioxide

Aerogels, resulting from the drying of wet gels while preserving their intrinsic porosity, are commonly fabricated through the sol–gel method, modifying the gel’s molecular structure [80]. Typically, gels exhibit a porous structure strengthened via washing, with pores partially filled by an organic solvent such as ethanol. Drying under specific conditions may lead to collapsed structures, giving rise to xerogels or cryogels. Supercritical drying, employing compressed CO_2_ above the critical point, helps sustain the porous structure by eliminating capillary forces [81,82,83]. This single-phase process ensures effective extraction. The resulting dried gel, upon exposure to air, experiences CO_2_ exchange, undergoing transformation into an aerogel [80]. CO_2_ is a fluid that is normally employed in the drying of cellulose aerogels because of its reasonable critical point (304 K, 7.4 MPa) and the benefits of low cost and great safety.

Supercritical drying is distinguished by the two-way mass transfer of the liquid gel solvent CO_2_ into and out of the wet gel pores [84]. To begin, the drying is largely caused by high scCO_2_ dissolution in the liquid gel solvent, which results in an expanded liquid and spilling of the extra liquid volume removed from the gel network. Second, the amount of CO_2_ increases over time until supercritical conditions are reached for the fluid mixture in the pores, without any intermediary vapor–liquid transitions. Finally, the presence of supercritical fluid mixtures in pores with no liquid phases causes a lack of surface tension, which precludes pore collapse in the gel structure during solvent removal [84]. 

Water with a high surface tension might destroy a cellulose network’s delicate and extremely porous structure, which is generated during the drying process. The reasons behind this phenomenon include variances in the specific energies during the transitions between solid–liquid and liquid–gas phases, along with the generation of inward forces near the solvent menisci along the capillary walls. As a result, it is necessary to entirely replace the high-surface-tension water [85]. In an NMMO (N-methylmorpholine-N-oxide) solvent system, for example, while manufacturing regenerated cellulose aerogels, the cellulose gel requires re-priming with water, followed by either ethanol and acetone exchange or solely acetone exchange [86,87]. In the case of employing an ionic liquid as the solvent system, the cellulose gel necessitates an initial re-priming step with water, followed by subsequent acetone exchanges conducted repeatedly [49]. Ethanol exchange is a popular treatment for natural cellulose aerogels [88,89]. It has been demonstrated that cellulose solvent residues reduce drying efficacy [47]. Furthermore, the surface tension of various liquids, as well as the shaking involved during the re-priming and solvent exchange procedures, may destroy the cellulose gel structure [47,90]. The solvent exchange process is exceedingly slow, requiring an average of 2–3 days. Finally, supercritical drying using scCO_2_ can be helpful to reduce damage caused by capillary pressure inside the pores, which can be advantageous, as it promotes the production of aerogel materials with enhanced uniformity in their 3D network.

However, even though liquid CO_2_ is costless, because a high-pressure tank is required, this method is costly [11]. Nevertheless, the SCD processes offer a notable advantage in that the choice of solvent for the gelation process is highly versatile, allowing for a wide range of options. This approach is applicable across several types of gel materials and is not limited to specific ones. Two fundamental supercritical CO_2_ drying strategies exist: (A) high-temperature (HT) drying and (B) low-temperature (LT) drying [91].

(A) High-temperature scCO_2_ drying: After a pre-pressurization stage, the solvent is heated to above its critical point in the high-temperature (HT) process. This method requires heating the wet samples and solvent to supercritical temperatures in a sealed autoclave. For commonly used organic solvents, which have a critical point above 200 °C and a critical pressure ranging from 40 to 80 bar, the desired conditions can be achieved through this method. Subsequently, a slow depressurization is carried out [92,93,94,95,96]. The process and instrumentation involved in the high-temperature (HT) approach are straightforward, as it does not require pumps, and it enables direct surface modification to produce hydrophobic aerogels. However, one disadvantage of utilizing organic solvents is the risk of fire in the case of an unintentional or uncontrolled discharge, and the higher temperature may cause damage to heat-sensitive components. An alternative to the normal HT method is to drop the temperature slightly, maintaining it below the solvent’s critical temperature [97,98].

(B) Low-temperature scCO_2_ drying: Low-temperature drying uses supercritical carbon dioxide (CO_2_), since it has a low critical temperature (31 °C), is non-flammable, and is ecologically friendly. To eliminate all solvents, wet gel samples are periodically or continuously flushed with supercritical CO_2_. Heat exchangers and a liquid CO_2_ pump are critical equipment components for both periodic and continuous operations. The continuous low-temperature (LT) method may need a greater volume of CO_2_; nevertheless, particular drying costs can be greatly reduced via careful optimization and scaling to an industrial level [99,100]. Supercritical conditions offer the possibility of functionalizing the aerogel skeleton [101]. Considering the significance of aerogels in both scientific research and industrial applications, extensive studies have been carried out to examine the influence of drying conditions, diffusion, chemical composition, and temperature profiles on the quality of aerogels [80,81,82,83,102,103,104,105].

#### 2.2.2. Direct Vacuum Freeze-Drying and Freezing Facilitated by an Organic Solvent

Cellulose aerogels can be produced using a straightforward and environmentally friendly method known as vacuum freeze-drying. At a temperature below the freezing point of the liquid medium, which is usually water, the gel is initially frozen in this process. Much of the liquid is then eliminated through sublimation, which is an essential step to avoid structural collapse and reduce shrinkage. The implementation of freeze-drying can be done in two different ways: without solvent exchange, by using direct freezing; and by using organic solvent exchange. Direct freeze-drying is a process of low-temperature dehydration, involving freezing, primary drying, and secondary drying stages. Ice crystals, formed during the freezing phase, serve as templates for the structures of porous materials. During primary drying, amorphous ice crystals are sublimated at temperatures below the melting point of ice, by preventing the collapse of the pore structure. Subsequently, in the secondary drying phase, all ice crystals are removed, eliminating the bound water that is adsorbed on the material’s surface [106]. Consequently, the pore structure of porous aerogels, including their pore morphology and distribution, is influenced by the liquid crystallization process and growth behavior, which are controlled by the cooling rate and temperature. Additionally, various factors, such as cellulose content, gel size, shape, and temperature, affect the rate of sublimation, which is typically slow [11]. 

Another way of freeze-drying is to replace the solvent within the hydrogels with t-BuOH, which involves freezing and sublimation. Altogether, within the parameters mentioned above, freeze-dried aerogels that are made of nanocellulose and its derivatives are commonly encountered with a specific surface area, although the self-agglomeration of nanocellulose may reduce. For this reason, freeze-drying with an organic solvent exchange is another parameter that can affect the porous structure before drying. In particular, tert-butyl alcohol, which can be used instead of methanol or ethanol during solvent exchange, possesses a low interfacial tension and a single hydroxyl group, enabling it to create hydrogen bonds with the surface hydroxyl or carboxyl groups of nanocellulose and its derivatives [106]. Altogether, the presence of multiple butyl groups creates a steric barrier that inhibits the aggregation of nanocellulose. Consequently, when employed in solvent exchange, tert-butyl alcohol has the potential to preserve the gel structure of nanocellulose and its derivatives more effectively than water, thereby preventing the collapse of the cellulose aerogel structure [51,107,108,109].

By employing liquid nitrogen or liquid propane to enhance thermal conductivity, it is possible to quickly cool the cellulose gel. This process effectively reduces cellulose agglomeration and the formation of ice crystals, while simultaneously increasing the porosity of the resulting aerogel. Zhang et al. examined three different chilling rates in their study: liquid nitrogen (−196 °C for 30 min), a freezer with an extremely low temperature (−80 °C for 12 h), and a standard refrigerator (−20 °C for 24 h). Their observations revealed that the use of liquid nitrogen facilitated the rapid formation of ice crystals, thereby effectively mitigating cellulose self-agglomeration and leading to the development of a more homogeneous and seamless surface structure [110]. In order to achieve uniformly structured aerogels, the utilization of both anti-freezing chemicals [111] and spray freeze-drying techniques [112,113] depends on accelerating the freezing rate. However, before the advancement of the solid–liquid interface, comparable freezing rates and localized temperature gradients are observed, which is similar to the scenario of freeze-drying small samples in a freezer while simultaneously cooling the larger sample [11]. The specific surface area and pore size distribution of a given kind of cellulose aerogel are significantly influenced by the drying process employed [45,88]. Because of the creation of ice crystals and the high interfacial tension of water, freeze-drying typically generates fractures in the aerogel material. Other disadvantages of freeze-drying include its lengthy processing time and significant electric energy usage. In contrast, drying with supercritical carbon dioxide (scCO_2_) offers improved preservation of the cellulose gel structure, resulting in aerogels with minimal shrinkage, smaller pores, and higher specific surface areas [48,89,114,115].

#### 2.2.3. Ambient Drying

Atmospheric pressure drying of (ligno)cellulose aerogels is still in its infancy. The fundamental issue impeding the development of atmospheric drying for aerogels is significant network shrinkage produced through the liquid meniscus and pressure gradient. Under the same regeneration circumstances, vacuum-dried aerogels show significant shrinkage and collapse as compared to supercritical CO_2_-dried aerogels. According to ESEM images in the study referred to as [106], the capillary force during vacuum-drying degrades the porous structure [106].

The structure of cellulose aerogels may be adjusted and controlled using drying processes. For this purpose, four drying procedures are outlined. ScCO_2_ drying can result in mesoporous aerogels with large specific surface areas and high porosities. Although t-BuOH drying can achieve comparable results, the porous structure created is less homogeneous than that produced by ScCO_2_ drying [106].

## 3. Characterization Methods of Cellulose-Based Aerogels

Aerogels have two unique phases: the solid backbone and the pore phase, which are characterized by several essential characteristics (including phase proportion, the usual extent of each phase, and connectedness). Furthermore, the broad interface separating the two phases’ physical and chemical characteristics is one of aerogels’ crucial qualities [116]. Cellulose aerogels, as porous solid materials, possess qualities like those of other aerogels. Using cellulose in synthesis improves aerogels’ mechanical properties and moisture affinity. Chemical modification of cellulose for enhanced strength and structural characteristics is straightforward [11]. A preliminary assessment of an aerogel provides key information on its transparency, brittleness, deformability, sound upon impact (which indicates void and solid phases), organic components, and elastic modulus. Structural characterization methods offer more detailed insights [116].

### 3.1. Characterization of Cellulose Aerogels’ Structure

By applying structural characterization to cellulose aerogels, the rigidness, flexibility, or physical properties of the material can be determined, along with the component type (i.e., organic or inorganic) and how small or large its pores are [116].

The essential structural parameters of a porous material include the overall fraction of the porous and solid phases, the typical size/molecular structure of the backbone and porous phase, the connectivity between two phases, and the properties of the interface between them. These characteristics are evaluated using various characterization methodologies [116].

#### 3.1.1. Microscopic Analyses

Microscopic techniques offer valuable visual insights into the structure of aerogels at various length scales, including those as small as a few angstroms. While light microscopy (LS) has a resolution boundary of around 500 nm, it fails to capture the properties of most aerogels due to their particle sizes typically being less than 1 mm. Atomic force microscopy (AFM) theoretically has the potential to be used for aerogels but proves to be challenging in practice due to the irregular and deep surface topology found in fractured aerogels [116].

**Figure 3:**
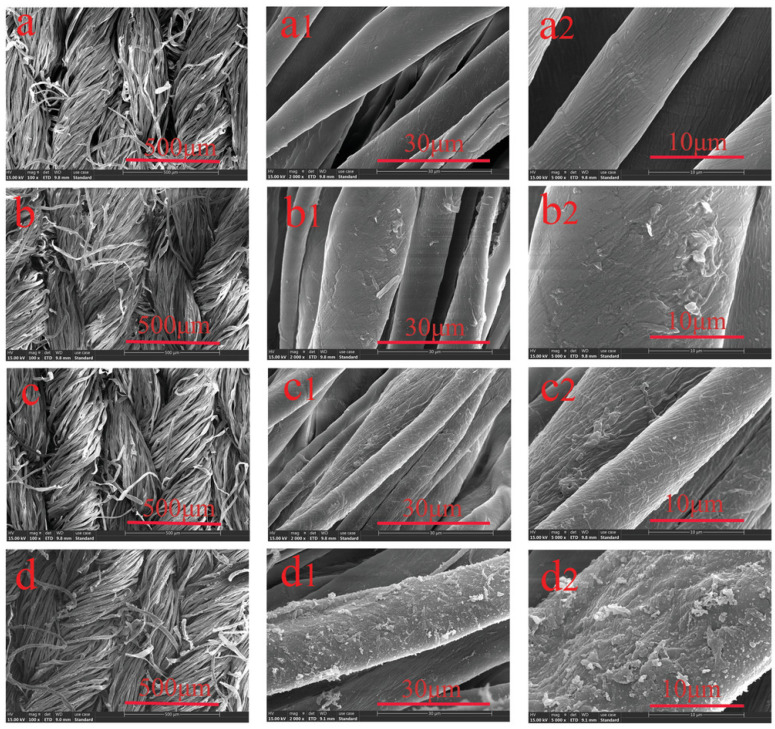
SEM images of various cotton samples: (**a**) untreated cotton (100), (**b**) cotton loaded with TA, (**c**) cotton loaded with TA/B, and (**d**) cotton coated with TA/B@PDA. The images were taken at different magnifications, specifically (**a**–**d**) 100, (**a1**–**d1**) 2000, and (**a2**–**d2**) 5000 (permission was approved by John Wiley and Sons, *Macromolecular Materials and Engineering*, under license number 5570770529195) [117].

Consequently, scanning electron microscopy (SEM) and transmission electron microscopy (TEM) are the primary microscopic techniques employed in aerogel research. SEM allows for the visualization of the three-dimensional interconnected structure of the aerogel’s backbone, with a maximum resolution of approximately one nanometer. In contrast, TEM has better resolution capabilities and can examine the substructure of the particles that make up the backbone, as well as any other phases employed in the aerogel skeleton, like metal particles. The released electrons and X-rays inside the hemisphere of the main beam are examined using a reflection setup in SEM mode. TEM, in contrast, investigates the sample’s transmission by high-energy primary electrons. All high-resolution electron microscopes operate with a chamber vacuum of less than 10^−4^ mbar to reduce undesired scattering [116]. The morphological and structural alterations of the cotton fiber, both before and after modification, as seen by SEM examination carried out at different times, are shown in Figure 3.

#### 3.1.2. Scattering Techniques

Scattering techniques provide a quantitative and noninvasive means of analyzing the structure without causing damage to the sample [118]. Moreover, they have proven to be valuable techniques for assessing the transparency of aerogels, investigating their pore size, structure, and mechanical strain, as well as exploring the sol–gel evolution modes that shape their microstructure. The examination of wavelength-dependent scattering enabled by ultraviolet–visible transmission spectroscopy permits comparisons across aerogels of various sources and thicknesses, as well as analyzing the influence of residual pollutants. Infrared reflectance tests provide the actual real and hypothetical refractive indices of porous aerogel materials, allowing researchers to better understand material characteristics and radiant heat transport. Scattering measurements at a given angle are useful for quality control, locating scattering sources, and evaluating inhomogeneities [119].

The dispersed intensity of aerogels often displays radial symmetry, suggesting an isotropic material. As a result, in a certain experimental arrangement, the angle θ becomes the only variable. However, if the aerogel sample has anisotropic qualities (due to drying conditions, for example), anisotropy will be seen in the scattering pattern, necessitating the use of directional information to explain the orientation of the anisotropy. In general, scattering is classified into two types: small angle scattering (SAS) and wide-angle scattering (WAS). When employing a reflection setup, X-ray scattering is often referred to as X-ray diffraction (XRD). SAS employs scattering angles (2θ) ranging from 0.001° to nearly 10°, with wavelengths between a few angstroms and nanometers. The equivalent q-range is 5 to 10^−4^ nm^−1^, with the lowest q-limit observed in ultralow-angle scattering (USAXS and USANS) [119,120,121].

#### 3.1.3. Thermoporometry

Thermoporometry (TPM) is a technique that examines the freezing behavior of a liquid within pores, which is achieved by observing the shift in the melting point of a liquid trapped within a mesoporous medium, compared to the surrounding free liquid [115]. By analyzing the resulting data, it is possible to extract details regarding the arrangement of pore sizes and the morphology of the pores. This technique is particularly sensitive to pores within the range of 2 to 30 nm. Unlike traditional porosimetry methods that involve mercury or gas adsorption and require dry samples, thermoporometry can be applied to gels, eliminating the need for sample drying; also, inorganic silica or cellulosic aerogels can undergo TPM in water due to their inherent hydrophobic nature [116,122]. This is a proposed method that offers a different approach to characterizing the structure of porous aerogels. It depends on the utilization of the Gibbs–Thomson equation, which was introduced by Gibbs in 1928 and Thomson in 1872. This equation measures the experimental shift in an interstitial liquid’s melting point caused by confinement within microscopic pores [123,124]. Thermoporometry extends its coverage to the microporous region, leading to a generally higher average pore size in aerogels compared to nitrogen sorption (BJH) [115]. Thermoporometry is anticipated to have a broader range of applications compared to gas sorption, as it encompasses pore sizes exceeding 50 nm. This method has successfully detected pores up to 430 nm in size [125]. The primary distinction between the two approaches lies in the fact that the gas adsorption method is carried out isothermally, varying the pressure on the dry sample. In contrast, TPM is observed isobarically, varying the temperature on the wet sample [126]. TPM is increasingly favored for determining porous materials’ PSD (pore size distributions) due to its quick analysis, minimal sample usage, and cost-effectiveness. It is advantageous for materials that are prone to collapse during drying, though it is less applicable to materials that are already dry, like aerogels. However, its non-traditional nature and less frequent use compared to gas adsorption or mercury porosimetry are drawbacks [122].

#### 3.1.4. Gas Sorption

The processed macro–mesoporous aerogels displayed the following characteristics concerning the flow of air passing through them in the atmosphere:

- Pressure–time curves were consistent with that of the theoretical model created for pure Darcy flow, which was employed to fit the data and determine the permeability constant.
- Permeability remained consistent regardless of the difference in pressure.
- The choice of surfactant had an impact on the permeability.

The abovementioned findings indicate that Darcy’s law may be used without considering other effects, such as Knudsen effects, the Klinkenberg effect, or slip flow at cell walls [127,128,129]. The most employed method for characterizing aerogels is nitrogen (N_2_) sorption at a temperature of 77.3 K. An extensive variety of relative gas pressures, ranging from 0 to 1, may be obtained by adjusting the gas pressure from vacuum to 0.1 MPa (1 bar). This method often yields useful data on specific surface areas as low as 0.01 m^2^/g and pore diameters ranging from 0.3 to 100 nm [116].

#### 3.1.5. Hg Porosimetry

Porosimetry using mercury (Hg) is a technique capable of investigating a broad range of accessible pore sizes, spanning approximately six orders of magnitude. This range extends from about 400 mm down to a few angstroms [130]. In theory, Hg porosimetry may be used to examine the distributions of pore sizes spanning between microns and nanometers. On the other hand, when working with flexible materials like aerogels, considerable deformation effects may arise unless the sample stiffness aligns well with the pore size [131,132]. One drawback of mercury porosimetry is that it frequently leads to irreversible deformation of the sample, and a portion of infiltrated mercury becomes captured. Consequently, the sample is rendered unusable for subsequent investigations and must be disposed of as toxic waste [116]. Consequently, a comparative study on pore size measurement of cellulose-based aerogels revealed no observable damage or degradation in the aerogel produced from softwood pulp cellulose nanofibrils [133].

### 3.2. Mechanical Characterization of Cellulose Aerogels

Thorough mechanical characterizations in assessing the suitability of aerogels for load-bearing applications are crucial to understanding their mechanical responses under operational conditions. Mechanical characterization studies must be meticulously constructed to reproduce or match the loading conditions met in the desired service environment. Subject to the diverse service conditions of aerogels, it is necessary to perform multiple types of loading tests to fully evaluate their mechanical responses.

These tests generally encompass compression, bending, tension, multiaxial stress states, and torsion under various loading circumstances, such as dynamic, fatigue, and quasi-static. The following approaches are routinely used in this regard [134].

#### 3.2.1. Differential Scanning Calorimetry (DSC) and Dynamic Mechanical Analysis (DMA)

DSC is a technique for measuring thermal effects during the heating of materials (e.g., glass transition temperature). This becomes particularly essential when aerogels incorporate viscoelastic elements, such as organic polymers. The relationship between the rigidity of aerogels and temperature is determined using DMA. In dynamic mechanical analysis (DMA), a sinusoidal load or displacement is applied, and the ensuing response is gauged. The data obtained are then scrutinized to ascertain the complex modulus or complex compliance. DMA can be performed under various modes, including tension, compression, bending, or torsion. DMA uses alternating loads at a constant frequency (generally 1 Hz) or at changing frequencies ranging from 0.1 to 20 Hz. The tests are carried out at temperatures ranging from −100 °C to 300 °C, with the setup for a three-point bending test. Dynamic mechanical analysis (DMA) findings display the loss and storage moduli as they vary with temperature. The peaks observed in the loss tangent as a function of temperature can serve as indicators for identifying the glass transition temperatures [135].

#### 3.2.2. Tension and Compression

Aerogels are not well suited for conducting impact tests; hence, the approach used to quantify strength may be problematic from an engineering standpoint. Bending or tensile testing is preferable. The test should be tailored to the material [136]. Furthermore, determination of the compressive stiffness of aerogel composites shows the microstructure’s effectiveness in transmitting applied stress, which is regulated by the solid content. The density of the composite increases with higher solid content, and this results in higher stiffness [137]. Aerogels’ elastic characteristics are commonly assessed using sound velocity measurements [138,139,140] or static methods [141,142,143,144]. It is well accepted that silica and cellulosic aerogels are “fragile materials” because of their poor mechanical properties, caused by the network’s limited connectivity and large porosity. The existence of defects, which act as stress concentrators, has a significant impact on rupture resistance [143,144,145,146,147,148]. Dog-bone-shaped specimens (as per ASTM D638 [149]) are often used for tension testing. Similarly, compression testing may be performed on cylindrical specimens (as per ASTM D695 [150]). It is critical to prepare the specimens’ surfaces so that they are smooth and free of flaws. The end surfaces of cylindrical compression specimens should be parallel to one another. To avoid mistakes, compression tests should be carried out on compression plates with extremely parallel surfaces, especially for brittle aerogels. To achieve adequate alignment, a frequently employed method is the utilization of a self-aligned compression fixture. It is necessary to calculate the entire stress–strain relationship, including the point of ultimate failure, at exceedingly low strain rates to evaluate the energy absorption capacity and strength, as represented by the area under the stress–strain curve. To assess the dimensional stability of samples, conducting constant deflection–compression set tests (referred to as Test-D in the ASTM D3574 standard [151]) is feasible. This test involves measuring the changes in the thickness of cylindrical specimens exposed to a range of compression strains at an elevated temperature for a specified duration [116].

### 3.3. Sound Absorption and Spreading of Cellulose Aerogels

Noise pollution, considered to be the second most significant environmental hazard impacting human wellbeing and living conditions after air pollution, necessitates the utilization of porous sound-absorption materials. These materials offer remarkable attributes like a broad range of sound-absorption frequencies, cost-effectiveness, and ease of shaping. With their voluminous porous structure, characterized by low density, high porosity, and extensive surface area, aerogels allow acoustic waves to enter deeply into their structure, enabling diverse interactions to occur [152,153,154,155].

**Figure 4:**
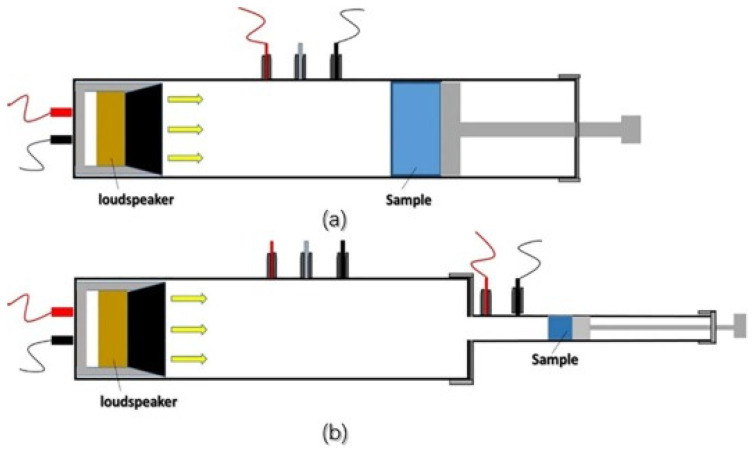
The schematic picture depicts the principle of measurement of sound absorption coefficients with an impedance tube at several frequency ranges. The frequencies in (**a**) vary from 50 to 1000 Hz, whereas the frequencies in (**b**) extend from 500 to 6300 Hz [159] (permission was granted by Creative Commons, CC-BY license).

Acoustic performance evaluations are often performed using an impedance tube (SW422, SW477, BSWA Technology Co., Ltd., Beijing, China), in accordance with ASTM E1050-10 [156]. The sound-absorption coefficient is expressed as α = (E_i_ − E_r_)/E_i_, where E_i_ denotes incoming acoustic energy and E_r_ denotes reflected acoustic energy. For sound absorption testing, samples with diameters of 100 mm and 30 mm have been used, with frequencies in the ranges of 200–1000 Hz and 1000–6300 Hz, respectively [157,158,159]. A demonstration was also carried out to evaluate the sound insulation capabilities of silica–cellulose aerogels and their cellulose matrices. The experiment used a sound signal generator (Blesi Guardian Angel anti-rape alert, 90 dB) to generate the known sound signals with a specified sound strength. The coefficient of absorption of sound for the silica–cellulose aerogels and the cellulose matrices was measured using a sound meter (Amprobe SM-10, Washington, DC, USA). The sound generator was positioned both inside and outside of an insulating box. The insulating box was built by taping a certain type of aerogel to both sides of the container, resulting in a sealed system. In all situations, the incident sound signal was measured at an equidistant position from the sound generator. The absorbed sound intensity was determined by subtracting the known sound intensity from the incident sound intensity. The coefficient for sound absorption was computed as the ratio of absorbed sound intensity to known sound intensity [160]. Figure 4 shows the principle of measurement of sound absorption coefficients with an impedance tube at several frequency ranges.

### 3.4. Thermal Characterization

Cellulose aerogels have great thermal insulation capabilities because of their exceptionally low thermal conductivity, making them ideal for insulation material applications. As the demand for thermal insulation materials continues to grow and evolve, traditional options like polyurethane and polystyrene foam face limitations due to their non-renewable properties. As a result, cellulose aerogels emerge as a groundbreaking, ecologically friendly substitute for old thermal insulation materials, while meeting the demand for long-term solutions in industry and society at large [161]. Thermal conductivity is widely recognized as the most crucial thermal property, with specific heat being of secondary importance [162,163]. 

The most frequent methods for investigating the thermal characteristics of cellulosic aerogels are thermogravimetric analysis (TGA), the transient plane source (TPS) method for thermal conductivity, and the derivative thermogravimetric analysis (DTG) transient hot-wire method [161,164,165,166].

Due to the chemical composition of cellulose aerogels, which consist of elements such as C, H, and O, they exhibit an elevated level of flammability. This characteristic imposes significant limitations on their use in various critical areas that necessitate flame-retardant materials. Consequently, it becomes imperative to pursue flame-retardant modifications for cellulose aerogels, aiming to enhance their fire resistance and broaden their applications [161]. Vertical burning tests (UL-94 [167]), limiting oxygen index (LOI) tests, and cone calorimetry tests are routinely employed to assess the flame retardancy of cellulose nanofibrils (CNFs) and their composite aerogels [164,165,166].

## 4. Properties of Cellulose Aerogels

Because cellulose-based aerogels have comparable porosity (84–99.9%), density (0.0005–0.35 g cm^−3^), and specific surface area (10–975 m^2^/g) to those of conventional silica aerogels and synthetic polymer-based aerogels, they have superior compressive strength (ranging from 5.2 kPa to 16.67 MPa) and enhanced biodegradability. Hence, new environmentally friendly and multifunctional materials called cellulose-based aerogels have been developed, showing immense potential for diverse applications. These applications encompass areas such as adsorption and separation of oil/water, thermal insulation, biomedical materials, carriers for metal nanoparticles/metal oxides, carbon aerogel production, and various other fields. The versatility of these materials opens numerous possibilities for their utilization. In Table 2, we have summarized aerogels’ main characteristic properties regarding their application field. As per Table 2, we can see that most of the properties are specified by their usage purpose and are seeing important improvements in the field. In Table 3, we mostly summarize the performance data of cellulose-based aerogels compared with other studies and their application areas. According to studies in Table 3, we can say that using textile fibers in cellulose aerogels increases their performance properties and makes them more qualified for diverse applications.

## 5. Multifunctional Application of Cellulose-Based Aerogels on Textile Structures

Due to the robust chemical reactivity of cellulose, the wide range of diverse derivatives with various functions, the adaptable construction process, and the multiple methods of modification, bio-based aerogels exhibit multifunctionality. There exist three primary methods for modifying cellulose aerogels [11]:

- Other components can be added to the cellulose solution/suspension [11]. For example, the reaction of CNF with N-methylol-dimethyphospylpropionamide (MDPA) and further crosslinking by 1,2,3,4-butane tracarboxylic acid (BTCA) yields a flame retardant with good flexibility and self-extinguishment [194].
- Coating or adding additional substances to the aerogel structure [11], such as the polyacrylonitrile–silica aerogel coating over viscose nonwoven fabric for protection and comfort [195]. Another area of study is the application of molecular layer-by-layer (m-LBL) technology. This technique enables the deposition of ultrathin layers onto a surface through sequential covalent processes. As a consequence, a precise molecular-scale coating is generated, mostly by surface oligomerization, which is not possible with bulk synthesis techniques [196,197,198].
- Surface modification of cellulose aerogels may be attained using a number of methods [11], including dip-coating with PDMS (poly(dimethyl siloxane)) [199].
- Cellulose aerogels are lightweight 3D porous materials. They are currently employed mostly in insulation, flame retardants [76,200,201], and biological applications [4,11]. Additionally, they find applications in carbon aerogel production, as well as the transportation of metal nanoparticles and metal oxides [11]. Therefore, the following sections will mostly discuss multifunctional applications in the textile field.

### 5.1. Thermal Insulation Materials

Materials are classified according to their thermal conductivity as thermal conductors (λ_eff_ ≥ 0.1 W/(mK), insulators (0.1 W/(mK) > λ_eff_ > 0.025 W/(mK), and superinsulators (λ_eff_ ≤ 0.025 W(mK). It is known that the thermal conductivity of dry air is around 0.025 W/(mK), which is generally slightly dependent on temperature and moisture content.

Due to their thermal conductivity levels spanning from tens to hundreds of W/(mK), metals are good thermal conductors. Expanded polystyrene, extruded polystyrene, glass wool, mineral wool, and wood exhibit thermal conductivities within the range of 0.1 to 0.026 W/(mK), making them effective insulators against heat transfer [163,202]. Silica aerogels, vacuum insulation panels, and vacuum glasses are regarded as superinsulating materials because their thermal conductivity is below 0.025 W/(mK) [203]. 

The thermal conduction of aerogels can be classified as solid-state, gas-phase, open-pore, or radiation thermal conduction. Once the pore size of a porous material approaches the average free path of the gas (which is approximately 70 nm when vented), the thermal conductivity of the substance decreases. This is attributed to the fact that the pores impede gas flow and restrict convection, thereby hindering heat transfer. The thermal conductivity of mesoporous cellulose aerogels primarily depends on two factors: solid-state thermal conduction and gas-phase thermal conduction. These factors, in turn, are closely associated with the aerogel’s density (determined by the initial cellulose concentration), the pore size distribution, and the surface structures of the aerogel material [11].

Regenerated cellulose aerogels possess a porous structure with a relatively higher fraction of large pores compared to other cellulose aerogels. This increased presence of large holes within the aerogel structure enhances heat conductivity as it facilitates improved gas transport [11].

**Figure 5:**
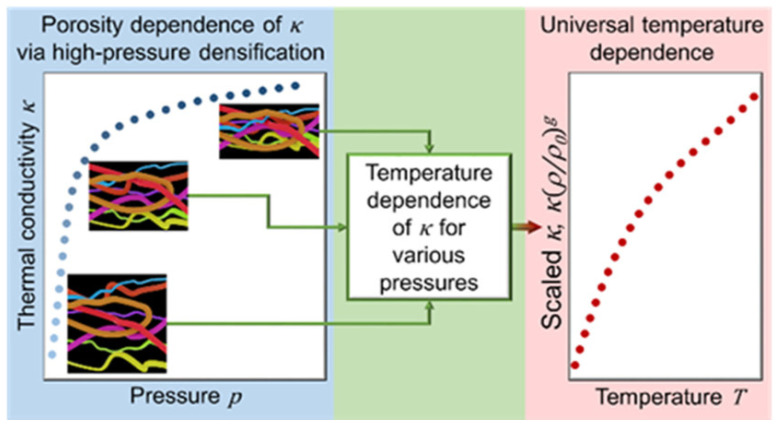
Thermal conductivity against pressure (note: this has been approved by ACS, and any further permissions connected to the content excerpted should be given to ACS) (https://pubs.acs.org/doi/10.1021/acs.biomac.1c00643 accessed on 10 October 2023) [204].

Antlauf et al. conducted a study in which cellulose fibers (CFs) and cellulose nanofibers (CNFs) were produced from commercially available birch pulp. The production process involved varying pressure and temperature parameters as experimental variables. For temperatures ranging from 80 to 380 °C, their results exhibited very little fluctuation in thermal conductivity with density (ρ_sample_ = 1340–1560 kg/m^−3^). Furthermore, temperature dependency is independent of fiber size, density, and porosity. Figure 5 depicts their studies on thermal conductivity [204].

Thai et al. studied the oil repellency and insulation properties of sugarcane fiber by sol–gel synthesis and using freeze-drying. According to their findings, increasing the sugarcane fiber content has a substantial influence on thermal conductivity ranging between 0.031 and 0.042 W/(mK) [188]. Table 4 also shows various types of cellulose aerogels, their thermal conductivity data and application areas.

### 5.2. Flame Retardancy

Aerogels with a lightweight composition derived from bio-based materials draw the attention of academics because of their exclusive properties, some examples of which include being environmentally conscious, sustainable, and possessing amazing thermal insulation effectiveness [212,213]. The fire-resistant clothing used for firefighting is a form of specific thermal protection clothing used by firemen during firefighting operations [214,215]. As a result, advanced flame-resistant and thermally insulating materials with exceptional performance are vital in thermal protective garments to safeguard firefighters. Para-aramid polymer is now used mostly in thermal protective gear as a material that provides flame retardancy due to thermal insulation of porous fabrics created from it [209,216]. Using the wet spinning procedure, Liu et al. [209] revealed that a lab-scale nanofibril Kevlar aerogel exhibited strong flame-suppressing properties, characterized by a comparatively slow combustion rate (0.013 cm/s) and the ability to extinguish itself.

**Figure 6:**
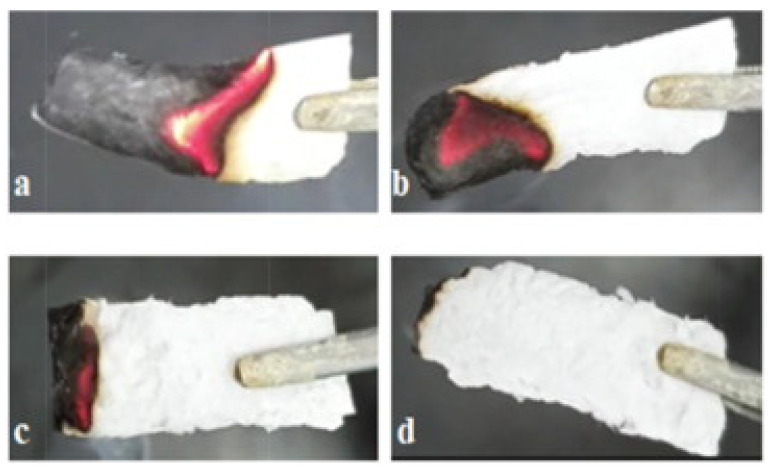
Images of a paper cellulose aerogel after a 10 s burn; the following samples were observed: (**a**) pure cellulose aerogel, (**b**) cellulose aerogel with 1% NaHCO_3_, (**c**) cellulose aerogel with 2% NaHCO_3_, and (**d**) cellulose aerogel with 3% NaHCO_3_ [223].

It is vital to identify environmentally acceptable thermal insulation materials designed for firefighting apparel [173]. Researchers have recently expressed interest in flame-retardant aerogels made from low-cost biomaterials because of their sustainable nature, eco-friendliness, affordability, lightweight properties, and strong thermal insulation properties [217,218,219]. Due to their high porosity, low thermal conductivity, lightweight structure, and excellent thermal insulation properties, aerogels find extensive utilization in various applications, such as fire resistance and thermal insulation [220,221]. Polymers derived from natural polysaccharides are common renewable biomass resources that are more biodegradable and environmentally friendly compared to fossil-based products [222]. In consequence, several efforts have been undertaken to create aerogels based on polysaccharides that exhibit remarkable low density, porosity, non-toxicity, biodegradability, and bio-sustainability [173]. Among the notable examples are magnesium hydroxide nanoparticles (MH NPs) in waste cotton fabric-based cellulose gel nanostructures [76] obtained by the freeze-drying method, which have demonstrated that the addition of magnesium hydroxide to the gel structure effectively enhances the flame-retardant properties of the aerogel in foam form. According to an experiment conducted by N. Le Thanh, adding NaHCO_3_ to the material showed a decrease in the combustibility of the material and the burning rate. Figure 6 shows this combustion example accordingly. As the figure and the experiments show, while the pure cellulose aerogel burned quickly (at an average speed of 3.45 mm/s), by increasing the NaHCO_3_ concentration in the material by between 1–2 and 3%, the combustibility of the material fell and its rate of burning also decreased [223].

### 5.3. Medical Applications

As the most common polymer on the planet, cellulose is mostly obtained from plants and microbiological sources [224]. Nevertheless, due to its unique properties, such as decomposability, compatibility with living systems, and low cytotoxicity, it is one of the most commonly used polymers for manufacturing aerogels [225]. Bio-based aerogels are widely employed in medical treatments such as biological detection, drug release systems, regenerative scaffolds, and anti-infective wound wrap materials [226]. Several studies have previously been published on the sequential evolution of aerogels’ formation and the therapeutic uses of nanofibrillated cellulose aerogels [17]. Nevertheless, little research has been conducted on the utilization of bio-based aerogels for bactericidal administration and wound treatment in textile applications. Many studies have explored strategies for wound healing, including the use of a composite aerogel with collagen and cellulose [227]. Collagen, which is valued for its adhesive, biodegradable, and biocompatible properties, proves suitable for wound dressings. Oxygen permeability and moisture management are crucial for normal cellular function in wound healing [228]. Cellulose-based nanoparticles, particularly nanocellulose polymers, leverage the effective surface area of filamentous biomaterials for cellular absorption. Combined with an antimicrobial substance [229], CNF aerogels emerge as a potential wound dressing solution. The antimicrobial efficacy of *Punica granatum* peels, surpassing conventional antibiotics, suggests their integration into aerogels for antimicrobial wound healing [230,231]. Medicinal applications of bio-based aerogels are listed in Table 5.

### 5.4. Water Treatment Containing Textile Dyes

Water pollution, a global concern affecting both water supplies and public health [234], involves contaminants like heavy metals [235], petroleum products, dyes, and various chemical compounds [236]. Ongoing efforts seek optimized methods for removing pollution sources, focusing on sustainable and environmentally friendly materials for water purification [234]. The water treatment method chosen depends on the water composition, quality criteria, and intended usage [237]. Iron removal is crucial for technical purposes to prevent water from becoming unsuitable due to high iron levels and discoloration. Dye release, a minor contributor to water pollution, is visible and undesirable even at low concentrations [236]. Annual dye production, surpassing 700 thousand tons, includes synthetic types posing risks to aquatic organisms and humans [238,239]. In textile manufacturing, dyes fall into categories such as anionic, cationic, or non-ionic [236]. Effluents from the dyeing process exhibit increased levels of color, suspended solids, biochemical oxygen demand, chemical oxygen demand, temperature, metals, and salts [240]. Continuous monitoring and comparison of these parameters with established concentrations are essential in treatment procedures before releasing effluents into water bodies. The assessment of treatment effectiveness also considers additional parameters such as total organic carbon, nitrate–nitrogen, ammonia–nitrogen, and orthophosphate–phosphorus [241]. Capturing dyes in fabrics during dyeing poses a challenge due to their pronounced water solubility, resulting in the generation of considerable wastewater with substantial quantities of these organic compounds [240]. The composition of wastewater in the textile industry exhibits global variations influenced by factors such as the manufacturing process, fabric type, factory equipment, applied chemicals, fabric weight, season, and fashion trends [241].

Various methods, including filtration, oxidation, and microbial approaches, are employed to eliminate dyes from water. However, these methods are associated with high costs, low efficiency, and operational challenges. Despite dyes’ resistance to degradation, certain bacteria, such as *Pseudomonas* sp. and *Sphingomonas* sp., have demonstrated effectiveness in decolorizing and mineralizing them [242]. The extensively used adsorption methods, employing traditional adsorbents like clay, bentonite, zeolite, or charcoal [10,11,12,13,243,244,245], are preferred for their cost-effectiveness, high efficiency, and simplicity. Nonetheless, traditional adsorbents face limitations such as a short effective life, regeneration difficulties, and high consumption. Color removal from wastewater involves a range of physical, chemical, and biological treatment methods [246], with adsorption widely acknowledged and employed [247].

Dyes in wastewater pose a significant threat to ecosystems and living organisms due to their persistence, biotoxicity, and bioaccumulation [248,249]. Consequently, there is a growing interest in developing efficient treatment strategies for dye effluents to address multipollutant removal [250]. Among the available technologies, adsorption is considered to be the most competitive, offering easy operation, relatively low costs, and non-toxic byproducts [251,252]. Cellulose-based aerogels are recognized as potential candidates for wastewater treatment due to cellulose’s renewable and biodegradable nature, easily functionalized properties, unique 3D network structure, and high surface area [246,253,254,255]. However, challenges remain, including avoiding toxic crosslinking agents and achieving a balance between high adsorption capacity and selectivity [246,256]. A green and effective strategy is essential to develop cellulose-based adsorption aerogel materials for dye wastewater purification [257]. Produced via the transesterification of cellulose and acetoacetate reagents, cellulose acetoacetate (CAA) is a water-soluble derivative that offers active reaction sites, which enable the creation of functional materials in aqueous environments [258,259,260]. In a noteworthy investigation, Liu and colleagues presented a self-repairing polysaccharide hydrogel formed by combining CAA and chitosan in a solution [261]. This hydrogel, derived from cellulose and employing enamine bonds, exhibited reversible sol–gel transitions that were responsive to pH variations. This highlights the suitability of polymers with amino groups for creating CAA-based gels. Unlike conventional chemical crosslinking agents, the eco-friendly approach of constructing 3D network structures with dynamic enamine bonds can be swiftly achieved at room temperature. The interlocking 3D network structure is also capable of being disassembled for further applications. Additionally, the use of electrostatic attraction, recognized as a simple and effective force, is emphasized for selectively capturing dyes, especially considering that the majority of commercial dyes exist in ion form [262]. In another study, a cationic cellulose aerogel (Q-CNF), derived from cellulose nanofibrils with trimethylammonium chloride groups, was prepared through freeze-drying and aliphatic triisocyanate crosslinking. The rigid porous aerogel demonstrated efficient adsorption of anionic dyes, with capacities of 250, 520, and 600 μmol g^−1^ (approximately 160, 230, and 560 mg g^−1^, respectively) for red, blue, and orange dyes, respectively. Electrostatic interactions between CNF surface positive sites and dye sulfonate groups were identified as the main contributors. The adsorption capacity was correlated with the specific surface area and cationic content of the aerogel. Regeneration with KCl in an ethanol–water mixture allowed for multiple adsorption–desorption cycles without significant capacity loss. Q-CNF aerogels exhibit promise as renewable, reusable adsorbents for treating dye-loaded water [263].

### 5.5. CO_2_ Capture

Carbon capture and storage (CCS) is a key component in the global effort to reduce carbon dioxide (CO_2_) emissions by preventing the release of CO_2_ into the atmosphere. Choosing the right materials is crucial to building dependable and secure infrastructure for CCS technology. Natural cellulose materials are a great option for CCS applications because of their impressive mechanical and physical qualities and eco-friendliness. As CO_2_ adsorbents and catalyst carriers for CO_2_ conversion, cellulose-based materials are useful in the field of carbon capture, utilization, and sequestration technologies [264]. Cellulose is a flexible material that may be used as a matrix or filler to produce goods such as films, paper bases, aerogels, and hydrogels with adsorption capabilities. It is known for its renewability and degradability [265,266,267,268]. Creating porous carbon with adsorption properties also requires it. Activation techniques employing physical or chemical activators can be used to modify carbon-based materials. Carbon capture technology appears to benefit from the use of cellulose and its derivatives, such as cellulose nanofibers (CNFs) and cellulose nanocrystals (CNCs), among other sophisticated materials [266,269,270]. Rich and affordable, cellulose aerogels have the potential to revolutionize existing carbon capture techniques, especially when it comes to the manufacturing of commercial nanocellulose [271,272,273]. Notwithstanding their smaller surface area, chemically altered cellulose and nanocellulose aerogels demonstrate adequate CO_2_ chemisorption, highlighting their potential for use in carbon capture applications [274,275]. By combining cellulose aerogels with large-pore hierarchical porous metal–organic frameworks (HP-MOFs), employing monocarboxylic acid (MA) as a modifier, and growing HP-UIO-66-NH_2_ on the cellulose aerogels in situ, Yu et al. created hybrid aerogels. The chain length of MA was changed to modify the pore size of the HP-MOFs. The study revealed that the CO_2_ adsorption capacity followed a trend of initially increasing and then decreasing with the increase in the MOFs’ pore size. Concurrently, the adsorption selectivity for CO_2_ consistently grew. Notably, among all of the samples, MC-HUN-4, distinguished by a moderate pore size, exhibited the highest CO_2_ adsorption capacity (1.90 mmol/g at 298 K and 1 bar) and superior adsorption selectivity (13.02 and 2.40 for CO_2_/N_2_ and CO_2_/CH_4_) [276]. Zhou et al. applied a sol–gel method to create composite aerogels (CSA) by using silica from skimmed cotton and cellulose whiskers. Employing tetraethyl orthosilicates (TEOs) and an alkaline silica solution as precursors, the CSA-TEPA 70% aerogel, with a 70% TEPA (tetraethylenepentamine) loading, demonstrated impressive adsorption, achieving a maximum capacity of 2.25 mmol/g. These adsorbents hold promise for CO_2_ capture. Additionally, the heightened research interest in metal–organic frameworks (MOFs) is attributed to their high surface area, CO_2_ affinity, structural diversity, tunable microporosity, and adaptable structure [277]. Using bacterial cellulose (BC) as a substrate, Ma et al. created composite aerogels containing amino-functionalized ZIF-8 (zeolitic imidazolate frameworks) (ZIF-8-NH_2_). Zinc ions and hydroxyl groups chelated to produce composites with strong interfacial attraction and compatibility when ZIF crystals were evenly encased around cellulose fibers. The resultant aerogel showed a noteworthy CO_2_ adsorption capability of 1.63 mmol/g. Zinc ions combined with the hydroxyl and oxygen groups in cellulose to create complexes. ZIF-8-NH_2_ crystals were created as linkers by adding 2-methylimidazole and 2-aminobenzimidazole by wrapping BC chains without the need for binders. By varying the amount of organic linker, the ZIF-8 amino group loading was optimized [278]. 

Mesoporous cellulose aerogels, derived from old corrugated containers (OCCs) through freeze-drying, exhibited efficient CO_2_ capture. The aerogel synthesis induced a transition in cellulose crystals from form I to form II while preserving their chemical structures. These aerogels featured excellent thermal stability, comprising highly porous networks with fibrils below 50 nm wide. Their mesopore volumes ranged from 0.73 to 1.53 cm^3^ g^−1^, and their specific surface areas varied from 132.72 to 245.19 m^2^ g^−1^. Furthermore, the research demonstrated exceptional CO_2_ adsorption capacities within the range of 1.96–11.78 mmol g^−1^. In comparison to other sorbents, the CA-2 material (2% weight cellulose) exhibited superior CO_2_ adsorption capacity at room temperature [279].

## 6. Companies Producing Cellulose Aerogels

Natural fiber aerogels with three-dimensional (3D) frameworks, light weight, a large surface-to-volume ratio, increased pore volume, and compatibility with living systems are generally used in ecological sediment accumulation [16,246], medical biology [280], and temperature isolation [281,282]. These characteristics make cellulose aerogels appealing for a variety of applications in a variety of sectors. Table 6 lists the current commercial cellulose aerogel providers.

## 7. Global Market Study Focused on Cellulose-Based Aerogels and Their Future Aspects

The aerogel market is witnessing significant growth driven by several factors, including the growing requirements of the oil and gas industry, along with the unique qualities of aerogels, such as exceptional heat resistance, recyclable use, and recoverability. In a recent report titled “Aerogel Market by Form (Blanket, Particle, Panel, and Monolith), Type (Silica, Polymers, Carbon, and Others), End-Use Industry (Building and Construction, Oil and Gas, Automotive, Aerospace, Performance Coatings, and Others): Global Opportunity Analysis and Industry Forecast, 2022–2032”, published by Allied Market Research, it was revealed that the global aerogel market reached a value of USD 1.3 billion in 2022. The commercial industry is projected to rise at a CAGR of 19.4% from 2023 to 2032, reaching a value of USD 7.5 billion. This growth is a result of the increasing applications of aerogels across various industries, including infrastructure development, petroleum, natural gas, vehicle manufacturing, aeronautics, efficiency-enhancing coatings, and others. The report highlights the diverse forms of aerogels, such as blankets, particles, panels, and monoliths, as well as several types including silica, polymers, carbon, and other materials. The forecast indicates a promising future for the aerogel market, driven by their wide range of applications and the growing demand for innovative and sustainable materials [289]. There is a significant current focus on the utilization of both synthetic polymers and biopolymers in the production of aerogels. Natural polymers derived from diverse reservoirs, including polysaccharides (such as sodium alginate, plant fibers, pectic substances, poly-(1,4)-2-amino-2-deoxy-β-D-glucan, poly-(1,4)-N-acetyl-D-glucosamine), lignocellulosic biomass, amino compounds, and other materials, have been employed as reactants for aerogel synthesis. For instance, the “Aerowood” project in the European Union aims to explore various fractions derived from lignocellulose, such as C5 and C6 sugars, for the purpose of aerogel manufacturing [290]. The obtained aerogels demonstrate a combination of the specific functionalities inherent to the utilized biopolymers and the characteristic traits of aerogels, including an open porous structure that has a high surface density and porosity. This synergistic blend of features presents a significant capacity for a broad range of utilizations. It is important to highlight that the characteristics of the biopolymers, such as their molar mass, components, and branching level, have a notable impact on both the overall properties of the aerogels and the molecular-level structure of their porous network [291].

**Figure 7:**
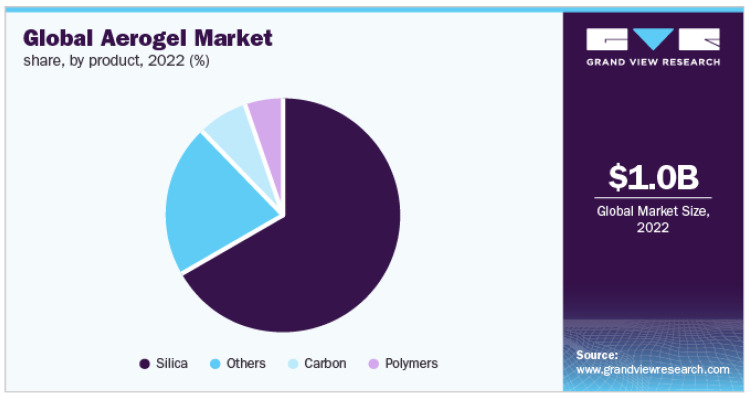
Global aerogel market size, including cellulose-based aerogels in the “others” and “polymers” [292] (note: This has been approved by the Grand View Search, and any further permissions connected to the content excerpted should be given to the Grand View Search) (https://www.grandviewresearch.com/industry-analysis/aerogel-market accessed on 16 August 2023).

Therefore, the primary focus of analysis in natural polymer aerogels revolves around defining numerical relationships between the characteristics of aerogels and the chemical nature of the raw materials, together with exploring various material mixes. Additionally, the ongoing search for alternative primary resources that offer an improved eco-friendly nature and cost-effectiveness is essential, particularly for applications with high demand [291]. Figure 7 shows the proportion of cellulose-based aerogels in the global aerogel market, along with the growth in general aerogel usage.

Cellulose aerogels hold immense promise for a range of prospective applications and advancements. The following are a few examples of prospects and potential breakthroughs linked to cellulose aerogels:

Eco-friendly insulation: A building’s thermal insulation is critical for reducing energy use and retaining an ideal indoor environment. Therefore, enhancing the thermal insulation properties of buildings is crucial, particularly by reducing losses of energy during heating and cooling applications, thus enabling energy savings. It is widely recognized that high-quality thermal insulation materials depend on various significant factors, including a reduced ability to conduct heat, renewability, cost-effectiveness, and environmental friendliness. Within this context, cellulose aerogels derived from biomass emerge as attractive materials that meet these criteria more effectively compared to conventional insulation materials [293]. Bio-cellulose aerogels have a few impressive features that make them excellent as thermal insulation materials. These properties include light weight [294], high porosity [295,296], large surface-to-volume ratio [297], low heat transfer [298], low heat expansion [299], high strength, high elastic modulus [300], flame retardancy [301], sustainability [302], and biocompatibility. These properties offer significant advantages in terms of providing long-term sustainable solutions for effective thermal insulation materials in newly developed applications [293].

Restoration of the environment: As well as the major applications of aerogels for thermal insulation in the aviation, space, and construction industries, they are also encouraging for other applications, such as the remediation of the environment, and major requisite features in the fields of materials and energy. Among these applications, environmental remediation stands out as a prominent area of focus. The field of aerogel-based environmental remediation has matured considerably and encompasses various air and water treatment processes. Aerogels are used in air cleaning for adsorbing CO2 from the atmosphere, along with eliminating pollutants such as volatile organic compounds from industrial and municipal effluents. Furthermore, they play a crucial role in water treatment by adsorbing heavy metal particles, oil, and toxic organic substances. These pollutants are essential contributors to the environmental challenges faced by our world today, including global warming and threats to human health [5]. There are many studies describing how to make adsorbents according to techniques for expediting the production process and enhancing the quality of bio-based materials derived from paper waste, employing a Kymene crosslinker to enhance the formation of gels rather than the alkali/urea dissolving agents and the lyophilization drying method. This mixed reclaimed fiber aerogel is highly hydrophobic and has a highly bending structure. According to the results, the maximum absorption capacity is 95 gg−1 at 50 °C with less than 1 wt% bio-based aerogel, due to its lower minimum density (0.007 g cm−3) and high maximum porosity (99.4%) [303]. Another investigation centered on the development of lightweight and hydrophobic watermelon carbon aerogels (WCAs), which exhibited remarkable selectivity in absorbing an extensive range of natural solvents and lubricants. These aerogels demonstrated an ultimate absorption limit ranging between 16 and 50 times their own weight, and they were able to maintain their absorption and harvesting capabilities over five cycles [304]. To extract anionic and cationic heavy metallic impurities from water, the synthesis of protein-infused carbon aerogels derived from cellulose (carbo gels) is successful for the capture of several risky agents like organic liquids, lubricants, and carbon dioxide, as marked by their sponge-like structure and tridimensional framework. These have lately piqued the interest of researchers for their potential in the cost-effective capture of heavy metals from waste and effluent streams [305]. The pore measurement, pore range, specific surface area, and structural chemistry are key properties that can be readily adjusted in aerogels to meet the specific requirements of adsorption uses. A diverse range of synthetic, natural, and blended aerogels have been proposed for this objective [306,307,308,309,310,311].

Energy storage: As modern society and the global economy continue to advance rapidly, the growing demographic-based consumption of exhaustible energy types like petroleum and methane has steadily risen. This has resulted in a pressing global challenge of depleting energy resources. Furthermore, the utilization of non-renewable resources unavoidably leads to environmental pollution [312]. To minimize the environmental repercussions and address the depletion of energy resources, there is an urgent necessity to develop state-of-the-art, affordable, and ecologically sustainable energy storage solutions [313]. Presently, two prominent energy storage technologies, namely, supercapacitors and rechargeable batteries, have garnered significant attention as highly promising options [314]. To produce high-performance electrochemical energy storage tools, effective electrochemical components are the main indicator [315,316]. It is necessary to improve the electrochemical behavior by designing the porous framework with a magnified, precise area for surface exposure and adjustable pore dimensions [317,318]. Likewise, for batteries, a larger precise area for surface exposure and appropriately sized pores create additional pathways for the migration of Li+ ions, resulting in increased volume. Thus, it is crucial to improve novel methodologies and renewable resources that can accomplish a substantial specific surface area while effectively controlling the sizes and volumes of the pores [319]. Because of the chemical and mechanical durability, superior resilience, and porous framework of the bio-based aerogels and foams, they exhibit excellent performance as structural reinforcement components for energy storage devices [312,320]. In contrast to conventional metallic support substances, foam structures and aerogels composed of cellulose offer distinct advantages in terms of lower density, improved flexibility, and enhanced electrochemical performance. These materials possess water-friendly surfaces and numerous absorbent locations, which promote the absorption and transport of electrolyte ions. Additionally, their structured pore hierarchy provides ample space for efficient power conservation [312,321]. For this reason, foam structures and aerogels derived from cellulose have gained recognition as prospective and environmentally sustainable configurations for combining with various other active materials in the modeling and production of cutting-edge energy conservation equipment, including energy-storing capacitors and reusable cells [297,321].

Medical applications: The appealing candidacy of bio-based aerogels in biomedical applications stems from their essential features of biocompatibility, biodegradability, and non-toxicity [322,323]. These bio-based aerogels, designed to mimic the extracellular matrices (ECMs) found within the body, have facilitated various biomedical applications. Examples include drug delivery [324,325,326], tissue engineering scaffolds [327,328,329], antibacterial agents [330,331,332,333], biomedical devices [334], biosensing platforms [335,336], and wound dressings [179,233,336,337].

Flexible electronic systems: Fibers and textiles play significant roles in various aspects of our daily lives. The incorporation of multifunctional elements into fabrics, particularly through utilizing nanoparticle investigations and informatics, is an expanding field of study. These materials can adapt to environmental changes or respond to external catalysts like mechanical, thermal, chemical, and magnetic effects [338,339,340,341,342]. Portable electronic devices and intelligent fabrics have evolved as new communication platforms with broad uses in industries as diverse as the medical sector, professional uniforms, sports, the power industry, and defense forces [290,291,292,293,294]. As a result, the development of compact and bendable wire-based electronic apparatus or fiber forms, as well as their incorporation into textiles, is gaining relevance [343,344]. Even though there have been many studies on aerogels regarding the flexibility of systems, cellulose aerogels show highly promising and effective results [345,346], such as the synthesis of holocellulose nanofibers/cellulose aerogel filaments using microscopic holocellulose fibers and a reformed cellulose structure from a water-based LiOH–urea solution. These holocellulose nanofibers/cellulose aerogel filaments present a novel procedure for a wide-ranging scope: the uninterrupted creation of eco-friendly, bendable, and durable aerogel strands with outstanding properties. To achieve this, the group combined specific pulping conditions, which help preserve the hemicellulose content, with mechanical defibrillation techniques, resulting in the fabrication of holocellulose nanofibrils [347,348,349,350,351]. These holocellulose nanofibers/cellulose aerogel filaments possess remarkable properties, including a significant dimension ratio, uniform measurements, outstanding physical strength, and exceptional ability to disperse [352,353]. Yamada et al. successfully created a bendable energy storage device by combining molybdenum electrical contacts, carbonaceous granules, and ionic fluid blends, alongside ionic gel conductive substances and bendable plant-fiber dividers. This assembled device exhibited excellent cycling stability, ensuring its long-term performance [354].

It is worth highlighting that investigations and progress in the domain of bio-based aerogels are currently in progress, and these upcoming facets signify promising avenues for further investigation. The anticipated progress and revelations are poised to unlock new applications and advantages for cellulose aerogels soon.

## 8. Conclusions

Since the turn of the century, cellulose-based aerogels have sparked a surge in technical and scientific interest due to their sustainable and ecologically favorable origins and 3D porous structure, which have gained them attention in adsorption, biomedicine, and thermal insulation. This review covers cellulose aerogels, discussing their production methods and limited applications in current textile practices, with a focus on their diverse functionalities in textiles. Despite their potential, the ongoing development of cellulose aerogels faces challenges in medical and environmental applications, particularly in the separation of water containing dyes. Current and future priorities include modifying cellulose aerogels to enhance their hydrophobicity, reduce their hydrophilicity, and address concerns about their toxicity and tissue compatibility. Overall, future efforts in functionalization will be crucial, and cellulose aerogels are expected to play significant roles in various applications, as mentioned in Section 7.

## Figures and Tables

**Table 1:** Classification of cellulose-based aerogel together with published examples.

Classification of Cellulose Aerogels						
Cellulose Aerogel Type	Starting Material	Solvent	Surface Chemistry	Drying Method	Application	Ref.
Natural cellulose	Pineapple leaf fiber, cotton waste fiber	Poly(vinyl alcohol) (PVA)	-	Freeze-drying	Building towards sustainable development	[31]
	Raw cotton fibers and cotton stalk	Tert-butyl alcohol	-		-	[59]
	Softwood cellulose pulp	TEMPO (2,2,6,6-tetramethylpiperidin-1-yl)oxyl)	Monocomponent endoglucanase, cupriethy lendiamine		Biofabrication of tissues, additional health and pharmacological uses	[60]
1.a. Nanocellulose	Cellulose nanofibers (CNFs), graphite powder, concentrated sulfuric acid, concentrated acetic acid, hydrogen peroxide solution	Sodium hydroxide, sodium hypochlorite, MO (methyl orange), and potassium permanganate	NaOH	Freeze-drying	The treatment of domestic organic wastewater	[61]
1.b. Bacterial cellulose	*Komagataeibacter sucrofermentans* H-110, TEMPO, dextrose, protein hydrolysate, yeast concentrate, disodium phosphate	Sodium hydroxide solution	NaClO, NaBr	Freeze-drying	Biofabrication of tissues and preparation of injury treatment materials	[4]
	Bacterial cellulose (BC) pellicles	-	Deionized water (DIW)		Pressure sensors, batteries and supercapacitors, substrates for catalysts, high-tech detectors	[62]
2. Regenerated cellulose	Cotton and viscose-based regenerated cellulose	Imidazolium acetate (EMIM), non-enium acetate ((DBNH)(OAc))	DMSO (dimethyl sulfoxide)	Supercritical CO_2_, lyophilization, ambitious drying	-	[63]
	Bamboo pulp boards	NaOH/urea aqueous solutions	Methyl-pyrrolidone (NMP), potassium hydroxide (KOH)	Freeze-drying	Application of energy storage devices	[64]
	Bamboo cellulose nanofibrils (BCNFs)	Polyvinyl alcohol (PVA)	Sodium tetraborate decahydrate (borax), N, N′-methylenebisacrylamide (MBA), methyltrimethoxysilane (MTMS)		Eco-friendly wrapping in the refrigerated transportation of fresh produce	[65]
3. Cellulose derivatives	Softwood kraft pulp sheets	1,2-Ethanediol, hydroxylammonium chloride monochloroacetic acid, poly-(1,4)-β-D-glucosamine	Sodium (meta) periodate, sodium chlorite	Freeze-drying	The production of advanced bio-adsorbents	[66]
	Softwood bleached kraft pulp (SBKP)	Water/tert-butyl alcohol	TEMPO-oxidized cellulose nanofibrils (TOCNs)		High-performance air filter	[67]
	Cellulose acetate	Acetone	Polymethylene polyphenylpolyisocyanate (PMDI)	ScCO_2_ drying	Thermal insulation applications	[52]

**Table 2:** Summarizing the characteristics of cellulose-based aerogels.

No	Aerogel Type	Main Properties	Distinctive Features	Application	Ref.
1	MXene composite aerogel (M−Aerogel)	Single-layered structure Conductive active material Three-dimensional porous structure Remarkable flexibility Superior compressive strength	- Up to 16 kPa compressive strength - 24,000 cycles of compression durability of the sensor - Up to 21.78 kPa pressure sensitivity	Flexible piezoresistive sensors	[168]
2	Holocellulose nanofibril (HCNF) aerogel from bamboo pulp and birch wood blocks	Fiber forms aerogel properties Exceptional self-cleaning capabilities Outstanding thermal insulation performance Washability Impressive tensile strength Biodegradability Superb mechanical properties Potential for weaving into multifunctional textiles suitable for demanding environments	- High specific surface area (413 m^2^/g) - High porosity (85%) - High strength (20.8 MPa) - Thermal insulation (−187 °C up to 190 °C) - 31.0% elongation at break	Thermal management and EMI shielding performance	[169]
3	Cellulose nanofibrils (CNFs) from rice straw cellulose	Amphiphilic–hydrophobic and oleophilic nature High porosity Extremely lightweight	- Ultralight (1.7 to 8.1 mg cm^−3^) - Ultra-porous (99.5–99.9%) - 96.8% yield from rice straw cellulose - 210 times water/375 times chloroform absorption	Selective oil removal and recovery	[170]
4	Barley–straw cellulose aerogels	Highly porous and lightweight aerogel, large surface area, high concentration of cellulose content	- Up to 30 times their mass storage capacity - Lowest density (0.0274 g/cm^3^) - Highest porosity (98.17%) - Largest surface area (49.5 m^2^/g)	Oil spillage cleanup	[171]
5	Bio-inspired tubular cellulose aerogel from kapok fibers	Exceptionally high compressive strength of 32 MPa, self-extinguishing capabilities, excellent flame retardancy, and cost-effective solution	- Ultrahigh compressive strength (32 MPa) - 13 μm diameter of tubular structure - Low thermal conductivity (0.054 W/mK) - Low density (41 mg/cm^−3^)	Exterior wall insulation and vehicle interiors	[172]
6	Bio-based aerogel (polysaccharide cryogel) from sodium alginate and chitosan	Eco-friendly and sustainable, excellent thermal insulation, bio-based flame retardant, ultralight porous structure, practical mechanical properties, and great flexibility, facilitating continuous flexing and rotating without fragmentation	- Low thermal conductivity (0.06–0.1 W/mK) - 11.3 kW/m^2^ radiant heat exposure - 10 kJ/g total heat release - 20 W/g peak heat release	Anti-flame apparel	[173]
7	Agar aerogels	Substantial surface area per unit weight, significant acceleration in wound healing in vivo, and the ability to be used for skin healing, in addition to its biocompatibility, renewability, and sustainability properties	- High porosity (97–98%) - High surface area (250–330 m^2^/g)	Wound dressings	[174]
8	Novel alginate–chitosan aerogel fibers	Highly porous structure reminiscent of cotton; non-cytotoxic, making it biocompatible; strong antibacterial activity, speeding wound closure; in vitro design imitating injured life-unit monolayer healing	- 162–302 m^2^/g specific surface area - 1.41–2.49 cm^3^/g specific pore volume	Wound-healing applications	[175]
9	Aerogels made of tempo-oxidized cellulose nanofibers and sodium alginate/chitosan	Serving as an interactive extracellular fabric derived from biological sources, and the capacity to degrade natural, highly porous structures, creating an ideal microenvironment for various applications	- 93% healing ratio - High-speed homeostasis (<6.0 s) - 98.99 ± 5.3 μm neoepidermis thickness - 2.65 ± 0.083 mm neoepidermis thickness	Wound dressings and injured tissue maturation	[176]
10	Alg–CaCO_3_ composite aerogels from sodium alginate	Cost-effective, environmentally friendly, ultralight, and fireproof, characterized by high permeability and excellent structural properties, reduced heat transfer rate, and excellent hydrophobic characteristics	- Up to 39.5% limiting oxygen index - Up to 92.40% porosity - Up to 0.936 MPa compression modulus - 0.031 W/mK thermal conductivity	Green fireproof building insulation materials	[177]
11	Kapok aerogel	Lightweight, providing insulation and robustness, reusable and decomposable, exceptional fire protection, high filling capacity, superior compressive resilience, and remarkable heat-insulating abilities	- 0.0531 W/mK thermal conductivity - 1.64 MPa compressive strength - 0.0587 g/cm^3^ minimum density	Application in emerging fields	[178]
12	Chitosan aerogel	Elevated permeability and extensive superficial expanse, enabling rapid local administration of antibiotics; infections are efficiently prevented early after wound debridement, while cell viability is maintained, absorbing substantial amounts of aqueous fluids	- Large surface area (>250 m^2^/g)—High porosity (>96%)	The management of chronic wounds	[179]
13	A novel intelligent bio-aerogel using cellulose/Salep/anthocyanins	Maintains structural integrity and allows for precise control over the porous structure; usage as intelligent aerogels in meat products, providing unique properties and benefits; serving as suitable matrices for pH-sensitive dyes, enabling their effective utilization	- 320 °C thermal decomposition - 13.5–16.1 mg/cm^3^ density of aerogels - 90.22 ± 0.02/91.61 ± 0.01% porosity - 17 ± 0.14/499 ± 9.90 mPa·s viscosity	Application in beef packaging	[180]
14	Essential-oil-loaded starch/cellulose aerogel	Aerogels with antimicrobial properties made from affordable materials	- 18.42/54.77 mg/cm^3^ density - 64 ± 0.01/87 ± 0.01% porosity - 24.73–95.5 µm pore size - 256 µL/L_headspace_/512 µL/L_headspace_/minimum inhibitory dose	Application in cheese packaging	[181]
15	Hybrid bio-aerogel with green pectin (PML) and corn stalk nanofiber (CNF)	High porosity and low density, providing excellent elasticity; exhibits a remarkable oil sorption capacity, ranging from 82 to 161 g/g.	- 82–161 g/g oil sorption capacity - At least 15 cycles of reuse - 99.9 μm pore size - 99.0% porosity - 5.3 mg/cm^3^ density	Applications in oil pollution treatment	[182]
16	Nanofibrillated cellulose/chitosan aerogel	Lightweight and flexible, with a well-defined three-dimensional linked cellular network structure, exhibiting outstanding mechanical properties both in air and underwater, a high maximum adsorption capacity, a rapid adsorption rate, and offering a low-cost solution with a long lifespan	- 197.33 and 134.12 mg/g maximum adsorption capacity - 752 mL purifying industrial wastewater - 49.71 mg/g preconcentration capacity - High regeneration efficiency (80.25%)	Heavy metal pollution in agriculture	[183]
17	Aerogels comprising graphene oxide (EGO) and TEMPO-oxidized cellulose nanofibrils (TOCNFs)	Great promise as an environmentally friendly conductive ink suitable for printing 3D objects using the direct ink writing (DIW) method; the inks exhibit a high yield stress, improved electrical conductivity, uniform distribution of micro- and nanoscale fibrils, and efficient penetration, representing a sustainable approach to produce conductive carbon-based inks	- 250–1096 kPa compression modulus - 55.6 dB EMI shielding effectiveness - Low concentrations from 2 to 5 wt% show high printing fidelity	Advanced applications (EMI shields)	[184]
18	Silica–cellulose nanoclaw hybrid aerogels	A biomimetic hybrid technique that is eco-friendly, cost-effective, and offers outstanding formability and mechanical stability, as well as substantial surface area per unit weight, strength, light weight, and minimal heat transfer	- 16/58 MPa mechanical properties - 30–630 m^2^/g surface area - 0.021 W/mK minimum thermal conductivity - ~33 LOI (limiting oxygen index) - 130 °C contact angle of aerogel	Structures, industrial production, air transport, and cosmic space	[185]

**Table 3:** Cellulose aerogels’ performance data in different application fields.

No	Cellulose Aerogel	Compressive Modulus	Thermal Conductivities	Adsorption/Absorption Capacity	Photothermal Effect	Flame Retardant	Applications	Ref.
1	Microcrystalline cellulose	1.4–16.2 MPa	0.04–0.075 W/mK	-	-	-	Textile and non-textile applications	[186]
2	Pineapple leaf and cotton waste fiber aerogels	11.33–44.63 kPa	0.039–0.043 W/mK	-	-	-	Insulation materials	[31]
3	Hybrid coffee–cotton aerogels	15.6 kPa	0.037–0.045 W/mK	16 g/g oil absorption	-	-	Thermal insulation, oil absorption, filtration	[187]
4	Biodegradable sugarcane bagasse aerogels	88 kPa	0.031–0.042 W/mK	25 g/g	-	-	Thermal insulation in building construction and oil cleanup in marine ecosystems	[188]
5	Micron-down feather fiber-reinforced cellulose composite aerogel	39.3–194.6 kPa	0.06012 W/mK	0.0991 sound absorption coefficient	-	-	Acoustic and thermal insulation	[189]
6	Bacterial cellulose/γ-(2,3-epoxypropoxy) propytrimethoxysilane trimethoxysilane composite aerogel (BK aerogel)	20.1 kPa	-	81 mg/g oil absorption	-	-	Diverse applications	[190]
7	Nanofibrillated cellulose-based aerogel	7.92 kPa	-	14.7–30.3 g/g absorption of oils and organic solvents	Higher absorptivity wavelength range (200–2500 nm)	-	Oil–water separation; high-viscosity and high-melting-point oil cleanup	[191]
8	Phosphorous/nitrogen-containing flame-retardant (P/N FR) aerogels	Flame-retardant/water-repellent agent: before = 52.54 kPa after = 136.34–170.91 kPa	33.2–40.3 mW/mK-	142 °C water contact angle	-	23% limiting oxygen index (LOI), UL-94V-0 grade	Thermal insulation	[164]
9	Phosphorus-containing flame-retardant modifying agent (DOPO-IA (9,10-dihydro-9-oxa-10-phosphaphenantherne-10-oxide)) aerogels	34.31–176.05 kPa	28.6–31.2 mW/mK	-	-	21–27% LOI	High-flammability applications	[161]
10	Regenerated cellulose/polyethyleneimine composite aerogel (RC/PCA)	371.78 kPa	-	980.39 mg/g methyl orange dye adsorption	-	-	Water purification	[192]
11	Green aerogels from rice straw	47 kPa	0.034–0.036 W/mK	13 g/g oil absorption /137.6–149.9 water contact angle	-	-	Thermal and acoustic insulation materials and oil absorbents	[193]

**Table 4:** Thermal insulation properties and application of cellulose-based aerogels.

No.	Material	Drying Method	Thermal Conductivity	Pore Size	Density	Application	Reference
1	Raw pineapple leaf fibers (PALF)	Freeze-drying	0.030–0.034 W/mK	1.38–2.21 nm	0.04 g/cm^3^	Heat and sound applications	[74]
2	Aerogels composed of bidirectional anisotropic polyimide/bacterial cellulose (b-PI/BC)	Freeze-drying	23–44 mW/mK (bidirectional PI/BC aerogels) 37–66 mW/mK (unidirectional PI/BC aerogels)	10–20 μm	46 mg/cm^3^	Practical and complex thermal insulation applications in buildings and aerospace	[205]
3	Aerogels made of fibrous silica and bacterial cellulose (BC)	Ambient pressure drying	-	13.7–15.5 nm	0.164 g/cm^3^	Wearable substances	[206]
4	Holocellulose nanofibrils/cellulose aerogel fibers (HCAFs)	ScCO_2_ drying	0.048 W/mK	265.4 ± 34.5 nm	0.22 g/cm^3^	Wearable substances	[169]
5	Multiscale nanocelluloses (NCs)	Freeze-drying	25.4 mW/mK	32–48 nm	7.2 kg/m^3^	Thermal insulation applications	[207]
6	Textile waste fiber (TWF) aerogel	Freeze-drying	0.049–0.061 W/mK	-	0.040–0.096 g/cm^3^	Building insulation and oil spill cleanup	[208]
7	Nanofibrous Kevlar aerogel threads	ScCO_2_ drying and freeze-drying	0.036 W/mK	11–12.8 nm	13 g/cm^3^	Thermal insulation and thermal management	[209]
8	Hydrophilic recycled cellulose aerogels	Freeze-drying	0.029–0.032 W/mK	40–200 µm	0.040 g/cm^3^	Sorption of water/oil, resistance of water, and thermal insulation	[210]
9	Silk fibroin aerogel	Freeze-drying	0.031 W/mK	19.71 ± 8.53	0.21 g/cm^3^	High-performance thermal insulation	[211]
10	Aerogels made of nanofibrillated cellulose	Spray lyophilization	0.018 W/mK	10 to 100 nm	0.012–0.033 g/cm^3^	Thermal superinsulating material	[112]

**Table 5:** Medical applications of cellulose-based aerogels and their applied methods.

Material	Drying Method	Applied Methods for Properties	Type of Obtained Aerogel	Application	Reference
- *Komagataeibacter sucrofermentans H-110* - *Hestrin and Schramm* - Sodium fusidate - NaBr - TEMPO	Freeze-drying	SEM, shrinkage of aerogels, porosity of aerogels, thermal conductivity, TGA, FTIR, antibacterial activity, AFM, cytotoxicity tests	Gel film (colorless, transparent)	Wound dressings	[4]
- *Populus ussuriensis* wood powder - Collagen - Sodium chlorite (analytical reagent) - Acetic acid - Potassium hydroxide (KOH) - Sodium hydroxide (NaOH) - Hydroxylamine hydrochloride (NH2OHHCl)	Freeze-drying	XRD, FTIR spectra, liquid substitution method, MTT assay	Powdered, dried, and ultrathin pellet	Wound bandages and biological tissue platforms	[227]
- MBGs (SiO_2_-CaO-P_2_O_5_-CuO) - Tetraethyl orthosilicate (TEOS), - Triethyl phosphate (TEP), - Calcium nitrate tetrahydrate - Copper(II) nitrate hemi(pentahydrate)	Ambient drying leading to self-assembly (EISA)	TEM, SEM, XRD, SXAS and N_2_ physisorption, stimulated body fluids (SBFs) in vitro, PCR analysis, Gram-negative bacteria, *Escherichia coli* (for antibacterial properties)	Fine powder (combined with the membrane structure which is obtained by the composite aerogel)	Chronic wound-healing dressings	[228]
- Cotton nanocellulosic crystal (CNC) - Sodium chloride (NaCl) - Potassium chloride (KCl) - Monosodium phosphate (NaH_2_PO_4_) - Hydrochloric acid (HCl) - Trifluoroethanol (TFE) - Sodium hydroxide (NaOH)	ScCO_2_ drying	Zeta potential for surface charge, circular dichroism (CD), X-ray diffraction (XRD), Rietveld method to determine crystallite size	-	Wound bandages and bactericidal activity	[232]
- Doxycycline hyclate - Alginate - Amidated pectin - Carbon dioxide (purity 99%) - Doxycycline	ScCO_2_ drying	SEM, sphericity coefficient (SC), UV–vis spectroscopy, encapsulation efficiency (EE), DSC, FTIR spectrophotometry, simulated wound fluid (SWF) contact	Core–shell gel droplets (beads)	Wound-healing process	[233]

**Table 6:** A list of current commercial cellulose aerogel providers [283].

Nation	Supplier	Chemical Composition of the Aerogel	Trade Name	Configuration of Aerogel	References
Spain	Technalia	Cellulose aerogels from wooden pulp	Inacell	Cellulosic sponge	[284]
Germany	Aerogel-it	Biomass and waste materials derived from agriculture, forestry, and marine ecosystems that are not intended for human consumption	Lignin aerogel	Boards	[285]
Estonia	Fibenol	Lignin, wood sugars, and specialty cellulose from wood residues	Lignova	Fine and coarse ground	[286]
Switzerland	Empa	TEMPO-oxidized nanofibrillated cellulose (NFC); chitosan	-	Monolith	[287,288]

## Data Availability

Not Applicable.

## Abbreviations

Acronym	Description
DP	Degree of polymerization
NaOH	Sodium hydroxide
NMMO	N-methyl-morpholine N-oxide
3D	Three-dimensional
PVA	Polyvinyl alcohol
TEMPO	2,2,6,6-Tetramethylpiperidin-1-yl)oxyl
CNFs	Cellulose nanofibers
MO	Methyl orange
NaClO	Sodium hypochlorite
NaBr	Sodium bromide
BC	Bacterial cellulose
DIW	Deionized water
EMIM	Imidazolium acetate
([DBNH][OAc])	Non-enium acetate
DMSO	Dimethyl sulfoxide
SC CO_2_	Supercritical carbon dioxide
NMP	Methyl-pyrrolidone
KOH	Potassium hydroxide
BCNFs	Bamboo cellulose nanofibrils
MBA	N, N′-methylenebisacrylamide
MTMS	Methyltrimethoxysilane
SBKP	Softwood bleached kraft pulp
(TEMPO)-(TOCN)	2,2,6,6-Tetramethylpiperidin-1-yl)oxyl, oxidized cellulose nanofibril
PMDI	Polymethylene polyphenylpolyisocyanate
PF	Pineapple fiber
CO_2_	Carbon dioxide
HT	High temperature
LT	Low temperature
ESEM	Environmental scanning electron microscope
t-BuOH	Tert-butyl alcohol
LS	Light microscopy
AFM	Atomic force microscopy
SEM	Scanning electron microscopy
TEM	Transmission electron microscopy
TA	Tannic acid
TA/B	Tannic acid/borax
TA/B@PDA	Tannic acid/borax polydopamine
SAS	Small-angle scattering
WAS	Wide-angle scattering
XRD	X-ray diffraction
USAXS	Ultralow-angle scattering
PSD	Pore size distribution
N_2_	Nitrogen
Hg	Mercury
NMMO	N-methylmorpholine-N-oxide
DSC	Differential scanning calorimetry
DMA	Dynamic mechanical analysis
SW422, SW477, BSWA Technology Co. Ltd., China	SW series impedance tubes can accurately measure sound absorption coefficients and impedance
Amprobe SM-10, USA	Sound Meter, United States of America
Hz	Hertz
CC-BY license	Creative Commons attribution
TGA	Thermogravimetric analysis
TPS	Transient plane source
DTG	Derivative thermogravimetric analysis
C, H, O	Carbon, hydrogen, oxygen
LOI	Limiting oxygen index
CNF	Cellulose nanofibril
MPa	Megapascal
HCNFs	Holocellulose nanofibrils
EMI	Electromagnetic interference
MXene	Two-dimensional (2D) layered conductive nanomaterial, composed of transition metal carbide/nitride
CaCO_3_	Calcium carbonate
PML	* Premna microphylla *
EGO	Electrochemically synthesized graphene oxide
DIW	Direct ink writing
TEMPO-(TOCNF)	2,2,6,6-Tetramethylpiperidine-1-oxyl oxidized cellulose nanofibrils
MDPA	N-methylol-dimethyphospylpropionamide
BTCA	1,2,3,4-Butane tracarboxylic acid
m-LBL	Molecular layer-by-layer
PDMS	Poly(dimethyl siloxane)
CFs	Cellulose fibers
PALFs	Pineapple leaf fibers
b-PI/BC	Bidirectional anisotropic polyimide/bacterial cellulose
HCAFs	Holocellulose nanofibrils/cellulose aerogel fibers
NCs	Nanocelluloses
TWFs	Textile waste fibers
MH NPs	Magnesium hydroxide nanoparticles
FTIR	Fourier-transform infrared spectroscopy
MTT assay	3-(4,5-Dimethylthiazol-2-yl)-2,5-diphenyl-2H-tetrazolium bromide assay
PCR	Polymerase chain reaction
SXAS	Small-angle X-ray scattering
CAGR	Compound annual growth rate
LiOH	Lithium hydroxide
